# Circumflex Coronary Artery Fistula Draining into Coronary Sinus

**DOI:** 10.21470/1678-9741-2021-0254

**Published:** 2022

**Authors:** Gabriela Carvalho Silva, Diana Patricia Lamprea Sepulveda Silva, Euclides Tenorio, Edmilson Cardoso dos Santos Filho, Fabiana Michelle Feitosa de Oliveira, João Henrique de Andrade Torres, Patrícia Eduardo Teles Correia, Clístanes Lucas Henrique Ferreira, Petrus Thiago Jorge Lopes da Silva, Fernando Moraes

**Affiliations:** 1 Cardiovascular Surgery Department, Instituto do Coração de Pernambuco, Recife, Pernambuco, Brazil.

**Keywords:** Fistula, Coronary Vessel Anomalies, Valvular Annuloplasty, Thrombosis

## Abstract

Coronary artery fistula draining into the coronary sinus is a rare vascular malformation, and its diagnosis and clinical manifestations usually occur late. We describe the case of a 72-year-old female patient with dyspnea on exertion (New York Heart Association Class III) associated with palpitations. The transthoracic echocardiogram showed significant tricuspid insufficiency. Cardiac catheterization showed aneurysm of the circumflex coronary artery and fistula of this artery draining into the coronary sinus. The patient underwent fistula ligation and tricuspid valve repair, with excellent surgical results.

**Table:** Abbreviations, Acronyms & Symbols

CAF	= Coronary artery fistula
CPB	= Cardiopulmonary bypass
TEE	= Transesophageal echocardiogram

## INTRODUCTION

Coronary artery fistula (CAF) is characterized by a communication of this artery with neighboring structures of lower pressure, and it rarely involves the circumflex artery, being more frequent from the left main coronary artery or the anterior descending coronary artery. In fact, CAF presents a very low incidence, even though it is the most common arterial malformation, being characterized as a rare cardiac anomaly^[1,2]^.

CAF treatment can be performed through surgical correction, especially when other structures will also be involved, or by transcatheter embolization. Such an anomaly must be repaired whenever the patient is symptomatic or asymptomatic, with continuous murmurs or systolic murmurs, and with an early diastolic component^[2,3]^.

Considering the low incidence of arteriovenous fistulas of the circumflex artery draining into the coronary sinus, we bring a case report of an elderly patient with an aneurysmatic circumflex artery fistula draining into the coronary sinus, associated with severe tricuspid valve insufficiency.

## QUESTIONS

- What is the relationship between coronary artery aneurysm and a fistula?
- Is there an association between CAF and severe tricuspid regurgitation?
- How is the CAF correction?
- Is there any association between coronary aneurysms and acute coronary syndrome?

### Discussion of Questions

A coronary artery aneurysm occurs when its diameter is > 1.5 cm and coronary atherosclerosis and inflammatory or connective tissue diseases are excluded. This fact occurs due to the high pressure between the coronary artery and the fistula outlet location, as in the case reported^[4]^.

Question A. Especially when there is connective tissue disease, there may be an aneurysm rupture, even in young patients, but it can also generate pericardial effusion of exudative content^[5,6]^.

Question B. The association between CAF and severe tricuspid regurgitation is rare and was recently reported in France, in 2016. When the flow from the coronary fistula draining into the venous sinus is very high, the jet can reach the tricuspid leaflet, thus generating or aggravating the insufficiency. In addition, the high flow of the CAF increases the volume load and thus causes atrial enlargement, dilating the tricuspid ring. There is also the dilation of the coronary sinus, caused by CAF, which can distort the anatomy of the mitral and tricuspid valve sinuses^[7,8]^.

Question C. Coronary fistula could be corrected by continuous or separated stitches. Generally, when it presents a high flow, it is possible to correct it with separate stitches. In special situations where the orifice is very large, there may be need to use larger grafts for its occlusion. In cases of giant aneurysms — as in a case reported in Japan, measuring 4 cm —, the venoarterial connection was connected, awaiting thrombosis of the aneurysm^[9,10]^.

Question D. There are also reports of association between coronary aneurysms and acute coronary syndrome, since stasis or decreased blood velocity can lead to the formation of thrombi, with their embolization being responsible for occasional cases of acute coronary syndrome^[11,12]^.

## BRIEF CONSIDERATION OF THE CASE REPORTED

The case was a 72-year-old female patient (48 kg and 1.57 m), without comorbidities and without previous cardiac surgery, complaining of dyspnea on exertion (New York Heart Association Class III) associated with palpitations for three years, with recent worsening and requiring medication adjustment. On physical examination, she was in good general condition, and her respiratory system presented diminished vesicular murmurs in the bases bilaterally and crackles in the left base. Regarding her cardiovascular system, she had an irregular heart rhythm, with a systolic murmur in the lower left sternal border, in addition to elevated jugular venous pressure and severe V wave. Her abdomen was flaccid, depressible, painless, with a palpable liver 5 cm from the right costal margin. Her extremities were heated and without swelling.

The electrocardiogram showed atrial fibrillation rhythm, and the chest X-ray showed enlargement of the cardiac area, affecting the right chambers and causing pulmonary dilation. Renal function was preserved (urea, 48 mg/dL; creatinine, 1.05 mg/dL). Hemoglobin was 12.7 g/dL, hematocrit was 39.9%, platelets were 221,000/mm^3^, international normalized ratio was 1.11 unit, and activated partial thromboplastin time was 30.2 seconds.

The transesophageal echocardiogram (TEE) showed a 29-mm diameter aorta, a 42-mm left atrium, and a left ventricular ejection fraction of 69%. The volume of the right atrium was 86 ml/m2. Pulmonary artery with normal diameters (pulmonary artery of 24 mm). Mitral valve with slight thickening and slight retraction of the anterior leaflet, with moderate (functional) reflux. Tricuspid valve with total loss of coaptation of the leaflets, which were retracted, with marked insufficiency. Aortic valve with mild calcification of the leaflets and preserved valve opening. Pulmonary valve without abnormalities. Presence of patent foramen ovale (3 mm) with right-left flow. Pulmonary artery systolic pressure was 55 mmHg. A turbulent flow was attributed to the coronary fistula. Global systolic dysfunction of the right ventricle, with tricuspid annular plane systolic excursion of 14 mm. Mild pericardial effusion (7 mm), without hemodynamic repercussions.

**Fig. 1:**
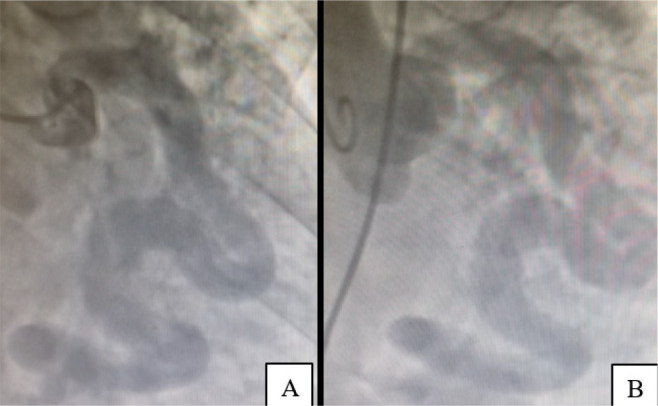
Circumflex artery aneurysm. (A) Coronariography. (B) Aortography.

Cardiac catheterization revealed a circumflex artery aneurysm with a fistula draining into the coronary sinus (Figure 1), as well as moderate venocapillary hypertension and mild pulmonary arterial hypertension. Coronary arteries were free of obstruction. Right ventricle with moderate diffuse hypocontractility and left ventricle with mild diffuse hypocontractility.

**Fig. 2:**
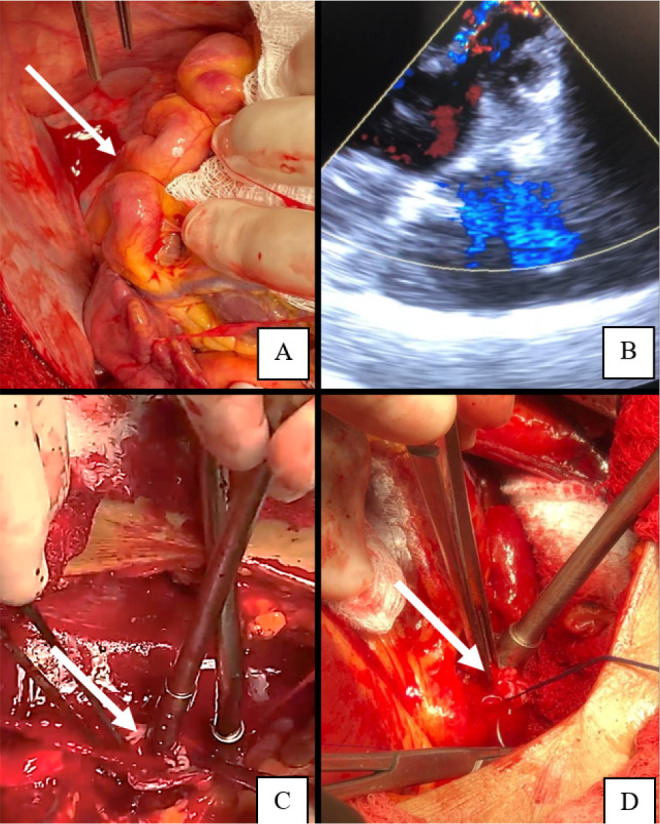
Aneurysmal fistula of circumflex coronary artery. (A) Surgical view. (B) Drainage into coronary sinus on transesophageal echocardiogram before correction. (C) Drainage site in coronary sinus fistula on surgical view. (D) Surgical correction.

The patient underwent surgery through median sternotomy and cardiopulmonary bypass (CPB) with mild hypothermia. Myocardial protection was performed with crystalloid St. Thomas cardioplegia infused at the root of the aorta. We found that the circumflex coronary artery was aneurysmatic and tortuous and had a mouth in the coronary sinus (Figure 2A). We performed TEE during the operation, which showed a high flow of the fistula (Figure 2B).

With the heart beating, the cardinal vein was incised longitudinally. At this point, it was possible to observe the communication between the circumflex artery and the coronary sinus. Then it was performed the fistula ligature with anchored 5-0 PROLENE™ sutures (Figures 2C and D).

The patient showed severe tricuspid regurgitation on the TEE image, however, when tested with saline solution during the operation, the valve had a small leak, so that for its correction only a slight reduction in the diameter of its ring was performed with ETHIBOND™ sutures anchored in pledgets.

After the cardioplegia administration, tricuspid valve annuloplasty was performed with 2-0 ETHIBOND™ stitches anchored in pledgets. The heartbeat was restored, and CPB was interrupted in good hemodynamic conditions.

When carrying out the control TEE, there was a small turbulent flow in the region of the coronary sinus. An additional 5-0 PROLENE™ suture was applied, and the fistula was completely ligated.

In the postoperative period, she was extubated in two hours and needed inotropic drug for three days. She presented mild acute kidney injury that progressively improved with conservative management. She was discharged on the 10th postoperative day.

The patient who participated in the referred study signed an informed consent form on the use of data from the clinical case and images of the surgery in scientific articles.

## LEARNING POINTS

- Surgical correction of the coronary fistula has good results.
- CAF correction should be indicated early whenever it is diagnosed.
